# Worm Domains are not Gromov Hyperbolic

**DOI:** 10.1007/s12220-023-01320-y

**Published:** 2023-05-31

**Authors:** Leandro Arosio, Gian Maria Dall’Ara, Matteo Fiacchi

**Affiliations:** 1 Department of Mathematics, University of Rome“Tor Vergata”, Rome, Italy; 2 Research Unit SNS Pisa, Istituto Nazionale di Alta Matematica “F. Severi”, Rome, Italy; 3 Faculty of Mathematics and Physics, University of Ljubljana, Ljubljana, Slovenia

**Keywords:** Worm domain, Gromov hyperbolicity, Kobayashi metric, Scaling, Primary: 32F45, Secondary: 32T20, 53C23

## Abstract

We show that Worm domains are not Gromov hyperbolic with respect to the Kobayashi distance.

## Introduction

A central problem in contemporary several complex variables is to determine when a complete Kobayashi hyperbolic domain $\Omega \subset \subset \mathbb {C}^n$ is Gromov hyperbolic when endowed with its Kobayashi distance. Assume in what follows that $\Omega$ is smoothly bounded.

Some families of relevant domains are Gromov hyperbolic: Balogh–Bonk [2] proved it for strongly pseudoconvex domains, and Zimmer [19] showed it for convex domains of D’Angelo finite type. The third-named author showed it [13] for pseudoconvex domains of finite type in $\mathbb {C}^2$. On the other hand, Gaussier–Seshadri [15] proved that for smoothly bounded *convex* domains $\Omega \subset \subset \mathbb {C}^n$ an analytic disk in the boundary is an obstruction to Gromov hyperbolicity. This result was later strengthened by Zimmer [19], who showed that the same is true if $\Omega$ is a smoothly bounded $\mathbb {C}$-*convex* domain. The following important question remains open.

### Question

Is an analytic disk in the boundary an obstruction to Gromov hyperbolicity for a smoothly bounded complete Kobayashi hyperbolic domain $\Omega \subset \subset \mathbb {C}^n$?

In this paper, we study the Gromov hyperbolicity of the Worm domains introduced by Diederich–Fornæss [11], which have a holomorphic annulus in the boundary and are highly non-$\mathbb {C}$-convex. Worm domains play a central role in several complex variables as they provide counterexamples to several important questions. See, e.g., [17] for a review of the properties of Worm domains. We actually consider a more general class of *Worms* (see Definition 10), with an open Riemann surface in the boundary, and prove the following result:

### Theorem 1

Worms are not Gromov hyperbolic w.r.t. the Kobayashi distance.

The proof is based on Barrett’s scaling (cf. [4, Sect. 4]). We rescale the Worm *W* obtaining in the limit a holomorphic fiber bundle, which we call a *pre-Worm*, with base an open hyperbolic Riemann surface and with fiber the right half-plane. We show that such a pre-Worm cannot be Gromov hyperbolic. Since the Kobayashi distance is continuous with respect to this scaling, this yields the result.

## Gromov Hyperbolicity—Basic Definitions

In this section, we will review some basic definitions and properties of Gromov hyperbolic spaces. The book [8] is one of the standard references.

### Definition 2

Let (*X*, *d*) be a metric space. For every $x,y,o\in X$ the *Gromov product* is

$$\begin{aligned} (x|y)_o:=\frac{1}{2}[d(x,o)+d(y,o)-d(x,y)]. \end{aligned}$$

The metric space (*X*, *d*) is $\delta$-*hyperbolic* if for all $x,y,z,o\in X$

$$\begin{aligned} (x|y)_o\ge \min \{(x|z)_o,(y|z)_o\}-\delta . \end{aligned}$$

Finally, a metric space is *Gromov hyperbolic* if it is $\delta$-hyperbolic for some $\delta \ge 0$.

### Definition 3

Let (*X*, *d*) be a metric space, $I\subset \mathbb {R}$ be an interval and $A\ge 1$ and $B\ge 0$. A function $\sigma :I\rightarrow X$ is

1. a *geodesic* if for each $s,t\in I$
  $$\begin{aligned} d(\sigma (s),\sigma (t))=|t-s|; \end{aligned}$$
2. a (*A*, *B*)-*quasigeodesic* if for each $s,t\in I$
  $$\begin{aligned} A^{-1}|t-s|-B\le d(\sigma (s),\sigma (t))\le A|t-s|+B. \end{aligned}$$

A (*A*, *B*)-*quasigeodesic triangle* is a choice of three points in *X* and three (*A*, *B*)-quasigeodesic segments connecting these points, called its *sides*. If $M\ge 0$, a (*A*, *B*)-*quasigeodesic triangle* is *M*-*slim* if every side is contained in the *M*-neighborhood of the other two sides.

Finally, recall that a metric space (*X*, *d*) is *proper* if closed balls are compact, and *geodesic* if any two points can be connected by a geodesic. A fundamental property of geodesic Gromov hyperbolic spaces is that quasigeodesics are uniformly close to geodesics, a fact which implies the following characterization of Gromov hyperbolicity.

### Proposition 4

[8, Corollary 1.8] A proper geodesic metric space (*X*, *d*) is $\delta$-hyperbolic if and only if for all $A\ge 1$ and $B\ge 0$, there exists $M\ge 0$ such that every (*A*, *B*)-quasigeodesic triangle is *M*-slim.

## Worms and Pre-Worms

Let *X* be an open Riemann surface, and let $\theta :X\rightarrow \mathbb {R}$ be a smooth “angle” function. Consider the domain in $X\times \mathbb {C}$ defined as follows:

$$\begin{aligned} Z(X, \theta ):=\{(z,w)\in X\times \mathbb {C}:\Re (we^{-i\theta (z)})>0 \}, \end{aligned}$$

which is readily seen to be a smooth fiber bundle with base *X* and fiber a half-plane.

### Proposition 5

If the function $\theta$ is harmonic, then $Z(X, \theta )$ is a holomorphic fiber bundle.

### Proof

Let *v* be (minus) a local harmonic conjugate of $\theta$, so that $F(z)=v(z)+i\theta (z)$ is a holomorphic function on an open set $U\subset X$. Then $Z(X, \theta )$ is locally defined over *U* by $\Re (we^{-F(z)})=\Re (we^{-v(z)-i\theta (z)})>0$, and $(z,w)\mapsto (z, e^{-F(z)}w)$ is the desired local trivialization. $\square$

### Definition 6

(*pre-Worms*) If the function $\theta$ is harmonic, we call the holomorphic fiber bundle $Z(X, \theta )$ a *pre-Worm*.

### Remark 7

Pre-Worms are sectorial domains in the sense of [5] (see in particular Example 2.2).

A pre-Worm $Z(X,\theta )$ with hyperbolic base *X* is complete Kobayashi hyperbolic by the following classical result.

### Proposition 8

([16, Theorem 3.2.15]) Let $\pi :E\rightarrow X$ be a holomorphic fiber bundle with fiber *F*. Assume that *F* and *X* are both (complete) Kobayashi hyperbolic. Then *E* is (complete) Kobayashi hyperbolic.

Now we proceed to the definition of the Worms. First of all, given two compact intervals $I,J\subset \mathbb {R}$ such that $I\subset J^{\circ }$, we denote by $\eta :\mathbb {R}\rightarrow [0,+\infty )$ any smooth function satisfying the following properties:

- on *I*, the function $\eta$ vanishes identically;
- on $\mathbb {R}\setminus I$, the function $\eta$ is real-analytic and satisfies $\eta ''>0$ (in particular, $\eta$ is strictly positive and $\eta '\ne 0$ on $\mathbb {R}\setminus I$);
- $$J=\{\eta \le 1\}$$.

The precise choice of a function $\eta$ satisfying the above properties is completely irrelevant for what follows.

Next, given an open Riemann surface *Y* equipped with a smooth angle function $\theta :Y\rightarrow \mathbb {R}$ and two compact intervals *I*, *J* as above, we define

$$\begin{aligned} W:=\{(z,w)\in Y\times \mathbb {C}:|w-e^{i\theta (z)}|^2<1-\eta (\theta (z)) \}. \end{aligned}$$

We assume the following:

- $\theta$ has no critical points where $\theta (z)\in \partial I$ or $\theta (z)\in \partial J$;
- $\theta ^{-1}(J)$ is a compact subset of *Y*.

### Proposition 9

The domain $W\subset \subset Y\times \mathbb {C}$ has smooth boundary. Moreover, if $\theta$ is harmonic, then *W* is Levi-pseudoconvex.

### Proof

The precompactness of the domain *W* is a consequence of our assumption that $\theta ^{-1}(J)$ is compact. The domain *W* has defining function:

$$\begin{aligned} r(z,w)=w\overline{w}-we^{-i\theta (z)}-\overline{w}e^{i\theta (z)}+\eta (\theta (z)). \end{aligned}$$

We show that $dr\ne 0$ for all $(z,w)\in \partial W$. If $\partial _{\bar{w}}r\ne 0$, this is clear. Since $\partial _{\bar{w}}r=w-e^{i\theta (z)}$ vanishes only if $w=e^{i\theta (z)}$, we may assume that this identity holds. Then necessarily $\eta (\theta (z))=1$, that is, $\theta (z)\in \partial J$, in which case

$$\begin{aligned} \partial _{\bar{z}} r=i\partial _{\bar{z}}\theta (z) we^{-i\theta (z)}-i\partial _{\bar{z}}\theta (z)\overline{w}e^{i\theta (z)}+\eta '(\theta (z))\partial _{\bar{z}}\theta (z) =\eta '(\theta (z))\partial _{\bar{z}}\theta (z)\ne 0 \end{aligned}$$

by our assumption about the critical points of $\theta$. This proves that *W* has smooth boundary.

Since Levi-pseudoconvexity is a local property, we may restrict the *z* variable to an open set $U\subset Y$ where $\theta (z)$ admits a harmonic conjugate *v*(*z*), as in the proof of Proposition 5. A local defining function for the boundary of *W* is then given by

$$\begin{aligned} e^{-v}r = |e^{-\frac{F}{2}}w|^2-2\Re (we^{-F})+e^{-v}\eta \circ \theta , \end{aligned}$$

where $F(z)=v(z)+i\theta (z)$ is holomorphic. Recalling that moduli squared (resp. real parts) of holomorphic functions are plurisubharmonic (resp. pluriharmonic), we see that $e^{-v}r$ is equal to a plurisubharmonic function plus $e^{-v}\eta \circ \theta$, which is a function of the variable *z* alone. If we show that the latter is subharmonic, we are done. One computes

$$\begin{aligned} \Delta (e^{-v}\eta \circ \theta ) = \Delta (e^{-v})\eta \circ \theta +2\nabla (e^{-v})\cdot \nabla (\eta \circ \theta )+ e^{-v}\Delta (\eta \circ \theta ), \end{aligned}$$

where $\Delta$ and $\nabla$ are the ordinary real Laplacian and gradient in $\mathbb {C}\equiv \mathbb {R}^2$. In *U*, we have

$$\begin{aligned} \nabla (e^{-v})\cdot \nabla (\eta \circ \theta )=-e^{-v}(\eta '\circ \theta )\nabla v\cdot \nabla \theta =0, \end{aligned}$$

by Cauchy–Riemann equations.

Next, notice that $e^{-v}=|e^{-\frac{F}{2}}|^2$ is subharmonic. Since $\eta$ and $e^{-v}$ are nonnegative, all we are left to do to check the nonnegativity of $\Delta (e^{-v}\eta \circ \theta )$ is to verify that $\Delta (\eta \circ \theta )\ge 0$. By direct computation, we see that

$$\begin{aligned} \Delta (\eta \circ \theta ) =4|\partial _{\bar{z}}\theta |^2 \eta ''\circ \theta , \end{aligned}$$

which is nonnegative thanks to our convexity assumption on the auxiliary function $\eta$. $\square$

### Definition 10

(*Worms*) If the function $\theta$ is harmonic (and satisfies the assumptions on page 3), we call the domain $W\subset Y\times \mathbb {C}$ a *Worm*.

**Fig. 1:**
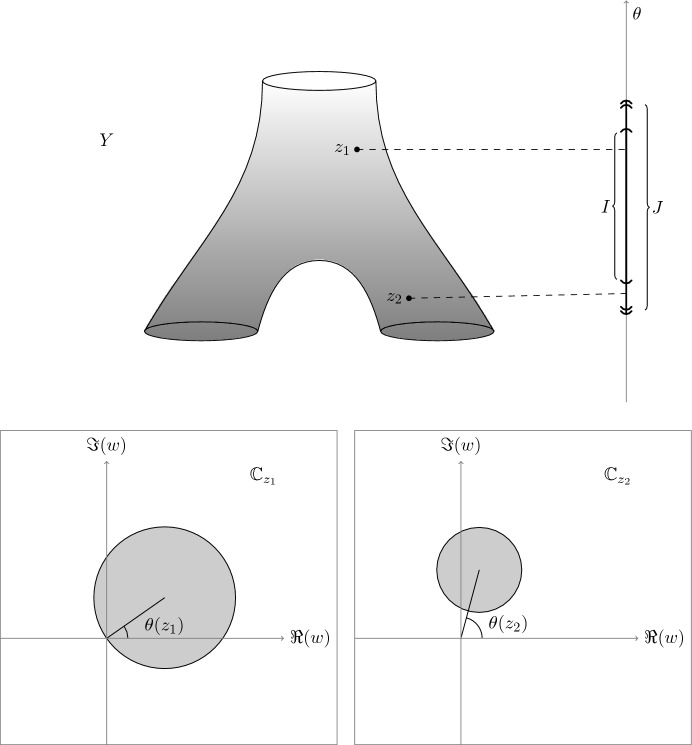
A Worm, whose underlying Riemann surface *Y* (depicted above) has genus zero and three boundary components. In this picture, the harmonic angle function $\theta$ is represented as a height function for visual clarity. In the two boxes below, one finds a generic *w*-slice of the worm over a point $z_1\in \theta ^{-1}(I)$ (on the left) and $z_2\in \theta ^{-1}(J\setminus I)$ (on the right). Notice that, because of the indicated choice of *I* and *J*, the surfaces $X_{\textrm{in}}$ and $X_{\textrm{out}}$ have the same topology (albeit in general different conformal structures)

The reader may find a picture of a Worm in Fig. 1.

### Remark 11

By Docquier–Grauert [12] every Worm is Stein.

For a more refined analysis, we split the boundary of *W* into four regions:

- the *spine* of the Worm
  $$\begin{aligned} S:= \{(z,w)\in \partial W:\theta (z)\in I, \ w=0\}, \end{aligned}$$
- the *body* of the Worm
  $$\begin{aligned} B:= \{(z,w)\in \partial W:\theta (z)\in I, \ \partial _z\theta (z)\ne 0, \ w\ne 0\}, \end{aligned}$$
- the *exceptional* set
  $$\begin{aligned} E:= \{(z,w)\in \partial W:\ \partial _z\theta (z)=0, \ w\ne 0\}, \end{aligned}$$
- the *caps*
  $$\begin{aligned} C:=\{(z,w)\in \partial W:\theta (z)\in J\setminus I, \ \partial _z\theta (z)\ne 0\}. \end{aligned}$$

### Remark 12

Identify the slice $\{w=0\}\subset Y\times \mathbb {C}$ with the Riemann surface *Y*. Inside *Y* the spine *S* is the closure of the domain

$$\begin{aligned} X_{\textrm{in}}:=\theta ^{-1}(I^\circ )\subset \subset Y.\end{aligned}$$

Since the angle function $\theta$ has no critical point $z\in \theta ^{-1}(\partial I)$, the domain $X_{\textrm{in}}$ is smoothly bounded. $X_{\textrm{in}}$ is a Riemann surface contained in the boundary of the Worm *W*; hence, every point of the spine *S* is of D’Angelo infinite type.

In what follows an important role is also played by the smoothly bounded domain

$$\begin{aligned} X_{\textrm{out}}:=\theta ^{-1}(J^\circ )\subset \subset Y.\end{aligned}$$

### Remark 13

The classical Worm domains introduced by Diederich–Fornæss [11] correspond to the case where $Y=\mathbb {C}^*$, $\theta (z)=\log |z|^2$. In this case, $X_{\textrm{in}}$ is a holomorphic annulus contained in the boundary of *W*, of which conformal class depends on the choice of the interval *I*.

A“genus zero”generalization of the Diederich–Fornæss Worms is obtained choosing $Y=\mathbb {C}\setminus \{a_1,\ldots , a_k\}$ and $\theta (z)=\sum _{j=1}^k\lambda _j\log |z-a_j|^2$ (where $\lambda _j>0$). If $I=[-a,b]$ with *a* and *b* large enough, the spine *S* has $k+1$ boundary components.

### Proposition 14

The caps *C* and the body *B* consist of strongly pseudoconvex points, the exceptional set *E* consists of finite-type points, and the spine *S* consists of infinite-type points.

### Proof

We already remarked that *S* consists of infinite-type points.

In the proof of Proposition 9, we saw that the boundary of a worm has a local defining function admitting the representation

$$\begin{aligned} \tilde{r}=|e^{-\frac{F}{2}}w|^2+e^{-v}\eta \circ \theta + \textrm{ph}, \end{aligned}$$

where $\textrm{ph}$ denotes a pluriharmonic function, and that

$$\begin{aligned} \Delta _z(e^{-v}\eta \circ \theta )\ge e^{-v} |\partial _{\bar{z}}\theta |^2 \eta ''\circ \theta . \end{aligned}$$

Since the latter quantity is positive on the caps (thanks to the strict convexity assumption on $\eta$), we conclude that the Worm is strictly pseudoconvex at every point of *C* where $\partial _{{w}}$ is *not tangent* to the boundary, that is $\partial _{ w}r\ne 0$ (or, equivalently, the vector (0, 1) is not in the complex tangent to $\partial W$). If instead $\partial _{ w}r= 0$, then we have $\theta \in \partial J$ (cf. the beginning of the proof of Proposition 9), and we may exploit the strong plurisubharmonicity of $|e^{-\frac{F}{2}}w|^2$:

$$\begin{aligned} \partial _{w}\partial _{\bar{w}}|e^{-\frac{F(z)}{2}}w|^2 = |e^{-\frac{F(z)}{2}}|^2>0. \end{aligned}$$

Thus, every point of *C* is strongly pseudoconvex.

We now study points (*z*, *w*) in the body *B*, where $\eta \circ \theta \equiv 0$. Calculating the Levi form $\mathcal L_{(z,w)}\tilde{r}$ we obtain, for $(a,b)\in \mathbb {C}^2$,

$$\begin{aligned} \mathcal L_{(z,w)}\tilde{r}(a,b)= \begin{pmatrix} \overline{a}&\overline{b} \end{pmatrix} |e^{-\frac{F(z)}{2}}|^2 \begin{pmatrix} |w|^2\frac{|F'(z)|^2}{4} &{} -\overline{w} \frac{\overline{F'(z)}}{2}\\ -w \frac{F'(z)}{2}&{} 1 \end{pmatrix} \begin{pmatrix} a\\ b \end{pmatrix}. \end{aligned}$$

Hence, $L_{(z,w)}\tilde{r}(a,b)$ vanishes if and only if $(a,b)\in \mathbb {C}^2$ is a multiple of $(2,wF'(z))$. This readily shows that the Worm is strongly pseudoconvex at every boundary point of the body where $(2,wF'(z))$ *is not in the complex tangent* to the boundary. But a simple computation shows that the vector $(2,wF'(z))$ is never complex tangent to the boundary since

$$\begin{aligned} (2\partial _z + wF'(z)\partial _w)\tilde{r} = wF'(z)e^{-F(z)}\ne 0. \end{aligned}$$

This shows that every point of the body *B* is strongly pseudoconvex.

We are left with the proof that every point of the exceptional set *E* is of finite type. By the Cauchy–Riemann equations, the critical points of $\theta$ are the same as the critical points of the (locally defined) holomorphic function *F*, and hence, they are isolated. Thus, *E* is a finite union of circles and circles with one point deleted (the point with $w=0$, in case the circle crosses the spine). Moreover, since $\theta$ has no critical points on $\partial I$, the boundary of the Worm is real-analytic in an open neighborhood of *E*. Thus, to verify that every point of *E* is of finite type, we need to check that no positive dimensional complex analytic variety lies in such a neighborhood (see, e.g., [3]). This is easy, because any point of such a variety would be of infinite type and, since we already checked that *B* and *C* consist of strongly pseudoconvex points, this would force the variety to be contained in *E*, which is impossible by dimension considerations (or by the open-mapping theorem). $\square$

### Remark 15

The Worms are examples of domains with nontrivial, yet nicely behaved, Levi core. See [9, 10], where this notion has been introduced by the second-named author and S. Mongodi. As a consequence of Proposition 14, the Levi core of a Worm is the $T^{1,0}$ bundle of its spine. A straightforward computation using [9, Proposition 4.1, part vi)] shows that the de Rham cohomology class on the spine *S* (or, equivalently, $X_{\textrm{in}}$) induced by the D’Angelo class of the Worm is represented by $i(\overline{\partial }-\partial )\theta$, which is exact if and only if the angle function $\theta$ is globally on $X_{\textrm{in}}$ the real part of a holomorphic function (that is, the pre-Worm $Z(X_{\textrm{in}}, \theta |_{X_{\textrm{in}}})$ is trivial as a fiber bundle). This is in turn equivalent to the condition that the Diederich–Fornæss index of the Worm is 1. We refer to [9, Sect. 4] for a review of the basic theory of D’Angelo classes and to [1, 9] for the implications on the Diederich–Fornæss index.

We end this section proving that Worms are complete Kobayashi hyperbolic. For this, we observe that a Worm *W* is naturally associated with two pre-Worms.

### Definition 16

Set

$$\begin{aligned} W_{\textrm{in}}:=Z(X_{\textrm{in}},\theta |_{X_{\textrm{in}}}),\quad W_{\textrm{out}}:=Z(X_{\textrm{out}},\theta |_{X_{\textrm{out}}}), \end{aligned}$$

where we are using the notation of Remark 12. Notice that $W_{\textrm{in}}\subset W_{\textrm{out}}$ and $W\subset W_{\textrm{out}}$.

In the remaining of the paper, if *M* is a complex manifold, we denote by $k_M$ its Kobayashi pseudodistance and by $K_M$ its Kobayashi–Royden pseudometric. The following lemma is proved in [14, Lemma 2.1.3].

### Lemma 17

Let $D\subset \mathbb {C}^d$ be a domain and $k_D$ its Kobayashi distance. If $z_n\rightarrow \xi \in \partial D$ and $\xi$ admits a local holomorphic peak function, then for every neighborhood *U* of $\xi$, we get

$$\begin{aligned} \lim _{n\rightarrow +\infty }k_D(z_n,D\cap U^c)=+\infty . \end{aligned}$$

### Proposition 18

Worms are complete Kobayashi hyperbolic.

### Proof

Assume by contradiction that there exists a nonconvergent Cauchy sequence $\{x_n\}_n$ in *W*. Passing to a subsequence, we can assume that $x_n\rightarrow \xi \in \partial W$. We write $x_n=(z_n,w_n)$ and $\xi =(z_0,w_0)$.

If $w_0\ne 0$, then $\xi$ is a pseudoconvex finite-type point by Proposition 14. By [6] (see also [18, Sect. 4]), $\xi$ admits a local holomorphic peak function, and hence, it cannot be a Cauchy sequence by Lemma 17.

Assume next that $w_0=0$, so that in particular $\xi \in \partial W_{\textrm{out}}$. Since $W\subset W_{\textrm{out}}$, it follows that $\{x_n\}_n$ is also a Cauchy sequence w.r.t. $k_{W_{\textrm{out}}}$, which converges to $\xi \in \partial W_{\textrm{out}}$. This contradicts the completeness of the pre-Worm $W_{\textrm{out}}$ (Proposition 8 below). $\square$

## Holomorphic Fiber Bundles are not Gromov Hyperbolic

We recall a classical result from the theory of Kobayashi hyperbolic complex manifolds. If *M* is a complex manifold, we denote by $B_M(p,r)$ the $k_M$-ball of center *p* and radius *r*.

### Proposition 19

([16, Proposition 3.1.19]) Let *M* be a Kobayashi hyperbolic complex manifold. Let $p\in M$ and $R, \epsilon >0$. Then there exists a constant $C\ge 1$ depending only on $\epsilon$ such that

$$\begin{aligned} k_{B_M(p,3R+\epsilon )}(x,y)\le C k_M(x,y), \quad \forall x,y\in B_M(p,R), \end{aligned}$$

and thus, the metrics $k_M$ and $k_{B_M(p,3R+\epsilon )}$ are biLipschitz equivalent on $B_M(p,R)$.

The fact that *C* depends only on $\epsilon$ is not stated explicitly in [16, Proposition 3.1.19], but it is clear from the (first paragraph of the) proof. We will actually use this result in the following simplified form.

### Corollary 20

Let *M* be a Kobayashi hyperbolic complex manifold. Then there exists an absolute constant $C\ge 1$ such that

$$\begin{aligned} k_{B_M(p,4R)}(x,y)\le C k_M(x,y), \quad \forall x,y\in B_M(p,R), \end{aligned}$$

for all $R\ge 1$ and all $p\in M.$

We introduce the following definition.

### Definition 21

Let $\pi :E\rightarrow X$ be a holomorphic fiber bundle and $z\in X$. Then define

$$\begin{aligned} r(z):=\sup \{r>0:\ \text { the bundle trivializes over } B_X(z,r)\} \end{aligned}$$

Notice that $r(z)>0$ for every $z\in X$ if *X* is Kobayashi hyperbolic.

We can now prove the main result of this section. Recall [16, Theorem 3.1.9] that if *X* and *Y* are two complex manifolds then

1 $$\begin{aligned} k_{X\times Y}((z_1,w_1),(z_2,w_2))=\max \left\{ k_X(z_1,z_2),k_Y(w_1,w_2)\right\} ,\quad z_1,z_2\in X,w_1,w_2\in Y.\end{aligned}$$

### Theorem 22

Let *X*, *F* be non-compact complete Kobayashi hyperbolic complex manifolds. Let $\pi :E\rightarrow X$ be a holomorphic fiber bundle with fiber *F* and such that $\sup _{z\in X}r(z)=+\infty$. Then $(E,k_E)$ is not Gromov hyperbolic.

### Proof

We will construct a sequence $\{T_n\}_n$ of quasigeodesic triangles in *E* violating the definition of Gromov hyperbolicity. Let $\{z_n\}_n$ in *X* be such that $r_n:=r(z_n)\rightarrow +\infty$. We define

$$\begin{aligned} \Omega _n:=\pi ^{-1}(B_X(z_n,r_n/2)), \end{aligned}$$

and let

$$\begin{aligned} \Psi _n:B_X(z_n,r_n/2)\times F\rightarrow \Omega _n \end{aligned}$$

be a holomorphic trivialization. Let $q\in F$ be any point of *F*. Let $C\ge 1$ be the universal constant given by Corollary 20. Set $t_n:=\frac{r_n}{16C}$.

We construct the triangles in the following way. Since *X* and *F* are non-compact, for all $n>0$, we can find a geodesic of *X* denoted $\gamma _n:[0,t_n]\rightarrow X$ with $\gamma _n(0)=z_n$, and a geodesic of *F* denoted $\sigma _n:[0,t_n]\rightarrow F$ with $\sigma _n(0)=q$. Notice that $\gamma _n([0,t_n])\subset B_X(z_n,r_n/8)$, so by Corollary 20 the curve $\gamma _n$ is a (*C*, 0)-quasigeodesic w.r.t. the Kobayashi distance of $B_X(z_n,r_n/2)$ (we may assume that $r_n\ge 8$ for every *n*).

By (1) the curves $a_n(t)=(z_n,\sigma _n(t))$ and $b_n(t)=(\gamma _n(t),q)$ are respectively a geodesic and (*C*, 0)-quasigeodesic of $B_X(z_n,r_n/2)\times F$. Moreover, a simple computation shows that the curve $c_n:[0,2t_n]\rightarrow B_X(z_n,r_n/2)\times F$ defined by

$$\begin{aligned} c_n(t)={\left\{ \begin{array}{ll}(\gamma _n(t),\sigma _n(t_n)) &{}\text{ if }\ t\in [0,t_n]\\ (\gamma _n(t_n),\sigma _n(2t_n-t))&{}\text{ if }\ t\in [t_n,2t_n].\end{array}\right. } \end{aligned}$$

is a (2*C*, 0)-quasigeodesic of $B_X(z_n,r_n/2)\times F$. Indeed, $c_n$ is a geodesic w.r.t. the distance $k_X+k_F$ that is 2*C*-BiLipschitz to $k_{B_X(z_n,r_n/2)\times F}$ in $B_X(z_n,r_n/8)\times F$. Hence, the triangle $T_n$ with sides $a_n, b_n$ and $c_n$ is a (2*C*, 0)-quasigeodesic triangle in $B_X(z_n,r_n/2)\times F$. Notice $T_n$ is not $t_n$-slim because

$$\begin{aligned} k_{B_X(z_n,r_n/2)\times F}(c_n(t_n),a_n([0,t_n])\cup b_n([0,t_n]))=t_n. \end{aligned}$$

Now since $\Psi _n$ is a biholomorphism between $B_X(z_n,r_n/2)\times F$ and $\Omega _n$, the triangle $\hat{T}_n$ in $\Omega _n$ that is image of $T_n$ via $\Psi _n$ is again a (2*C*, 0) quasigeodesic triangle w.r.t. $k_{\Omega _n}$, and it is not $t_n$-slim.

Now the map $\pi :E\rightarrow X$ is non-expanding, so

$$\begin{aligned} B_E(\Psi (z_n,q),r_n/2)\subset \Omega _n. \end{aligned}$$

The triangle $\hat{T}_n$ is contained in $B_{\Omega _n}(\Psi (z_n,q),r_n/8)$, and hence, it is contained in $B_E(\Psi (z_n,q),r_n/8)$. By another application of Corollary 20, the distances $k_E$ and $k_{\Omega _n}$ are *C*-BiLipschitz in $B_E(\Psi (z_n,q),r_n/8)$, so $\hat{T}_n$ are a $(2C^2,0)$-quasigeodesic triangle not $(C^{-1}t_n)$-slim w.r.t. the distance $k_E$. It follows that *E* is not Gromov hyperbolic. $\square$

**Fig. 2:**
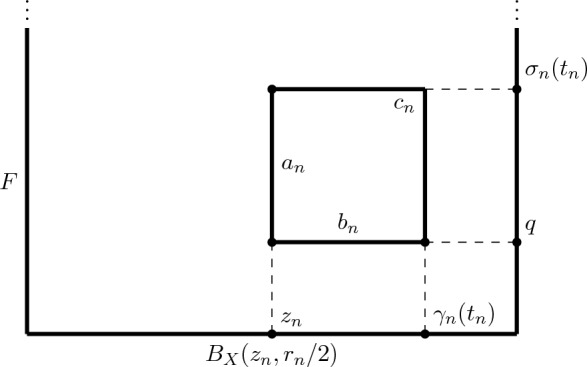
The triangle $T_n$ in $B_X(z_n,r_n/2)\times F$

We conclude this section highlighting an interesting class of holomorphic fiber bundles satisfying the condition $\sup _X r=+\infty$.

### Proposition 23

Let *Y* be a complex manifold and let $\pi :E\rightarrow Y$ be a holomorphic fiber bundle. Let $X\subset Y$ be a domain. Assume that there exists a point $\xi \in \partial X$ which admits a local holomorphic peak function. Then the restricted holomorphic bundle $E|_X$ has the property $\sup _X r=+\infty$.

### Proof

Let *U* be an open neighborhood of $\xi$ in *Y* such that $\pi :E\rightarrow Y$ trivializes over *U*. Let $\{z_n\}_n$ be a sequence in *X* converging to $\xi$. By Lemma 17, we have that

$$\begin{aligned} \lim _{n\rightarrow +\infty }k_X(z_n,X\cap U^c)=+\infty . \end{aligned}$$

Hence, for each $R>0$, we have $B_X(z_n,R)\subset X\cap U$ for *n* large enough, which implies that $r(z_n)\rightarrow +\infty .$ $\square$

### Corollary 24

The pre-Worms $W_{\textrm{in}}$ and $W_{\textrm{out}}$ are not Gromov hyperbolic w.r.t. its Kobayashi distance.

### Proof

The domains

$$\begin{aligned} X_{\textrm{in}}\subset \subset X_{\textrm{out}}\subset \subset Y \end{aligned}$$

are smoothly bounded (see Remark 12), and thus, every point in their boundary admits a local holomorphic peak function. Hence by the previous proposition the pre-Worms $W_{\textrm{in}}$ and $W_{\textrm{out}}$ satisfy $\sup _X r=+\infty$ and Theorem 22 yields the result. $\square$

## Barrett’s Scaling and Proof of Main Theorem

In what follows, we denote by *TM* the holomorphic tangent bundle of a complex manifold *M* and by $\pi :TM\rightarrow M$, the canonical projection. We denote by $\mathbb {D}\subset \mathbb {C}$ the unit disk. Recall the following classical definition.

### Definition 25

Let *M* be a complex manifold and let $X\subset M$ be a domain. Then *X* has *simple boundary* in *M* if for all $\phi :\mathbb {D}\rightarrow M$ holomorphic mappings with $\phi (\mathbb {D})\subset \overline{X}$ and $\phi (\mathbb {D})\cap \partial X\ne \varnothing$ one has $\phi (\mathbb {D})\subseteq \partial X$.

The proof of Theorem 1 is based on the following result, showing the stability of the Kobayashi distance and of the Kobayashi–Royden metric under a particular type of convergence of domains $D_n\rightarrow D_\infty$.

### Proposition 26

Let *M* be a taut complex manifold and let $\{ D_n\}_n$ be a sequence of domains of *M*. Let $D_\infty \subset M$ be a complete Kobayashi hyperbolic domain with simple boundary. Assume that

(i) if $\{ x_n\}_n$ is a sequence converging to $x_\infty \in M$ and $x_n\in D_n$ for all $n\in \mathbb {N}$, then $x_\infty \in \overline{D}_\infty$;
(ii) for every compact $H\subset D_\infty$, there exists *N* such that $H\subset D_n$ for $n\ge N$.

Then as $n\rightarrow +\infty$ we have $K_{D_n}\rightarrow K_{D_\infty }$ uniformly on compact subsets of $TD_\infty$, and $k_{D_n}\rightarrow k_{D_\infty }$ uniformly on compact subsets of $D_\infty \times D_\infty$.

See, e.g., [16, Chap. 5] for the notion of tautness. The idea of the proof of Proposition 26 is similar to [7, Theorem 4.3]. The proof is based on two lemmas, valid under the assumptions of the proposition.

### Lemma 27

For every $H\subset D_\infty$ compact and $\epsilon >0$, there exists *N* such that for all $n\ge N$ and for all $v\in \pi ^{-1}(H)$, we have

$$\begin{aligned} K_{D_n}(v)\le (1+\epsilon )K_{D_\infty }(v). \end{aligned}$$

### Proof

Set $r:=(1+\epsilon )^{-1}\in (0,1)$. Define $\widehat{H}\subset D_\infty$ as

$$\begin{aligned} \widehat{H}:=\{\phi (\zeta )|\, \phi :\mathbb {D}\rightarrow D_\infty \text { holomorphic},\ \phi (0)\in H,\ |\zeta |\le r \}. \end{aligned}$$

The set $\widehat{H}$ is compact. Indeed, let $\{z_n\}_n$ be a sequence in $\widehat{H}$, i.e., there exist $\phi _n:\mathbb {D}\rightarrow D_\infty$ such that $\phi _n(0)\in H$, and $|\zeta _n|\le r$ such that $z_n=\phi _n(\zeta _n).$ Since $\phi _n(0)\in H$ for all $n\in \mathbb {N}$ and $D_\infty$ is taut (by [16, Theorem 5.1.3]), we can assume that $\phi _n$ converges uniformly on compact sets to a holomorphic map $\hat{\phi }:\mathbb {D}\rightarrow D_\infty$ and that $\zeta _n$ converges to $\hat{\zeta }$ with $|\hat{\zeta }|\le r$. But then $z_n\rightarrow \hat{\phi }(\hat{\zeta })\in \widehat{H}$. This proves that $\widehat{H}$ is compact.

Now, for each $v\in \pi ^{-1}(H)$ let $\phi :\mathbb {D}\rightarrow D_\infty$ be such that $\phi (0)=\pi (v)$ and

$$\begin{aligned} K_{D_\infty }(v)\phi '(0)=v. \end{aligned}$$

Using property (ii), there exists *N* such that for all $n\ge N$, we have $\widehat{H}\subset D_n$, which implies that if $\phi _r:\mathbb {D}\rightarrow D_\infty$ is defined by $\phi _r(z):=\phi (r z)$ then $\phi _r(\mathbb {D})\subset \widehat{H}\subset D_n$. Finally, using the definition of the Kobayashi–Royden metric, we have

$$\begin{aligned} K_{D_n}(v)\le r^{-1}K_{D_\infty }(v)=(1+\epsilon )K_{D_\infty }(v). \end{aligned}$$

$$\square $$

### Lemma 28

For every $H\subset D_\infty$ compact and $\epsilon >0$, there exists *N* such that for all $n\ge N$ and for all $v\in \pi ^{-1}(H)$, we have

2 $$\begin{aligned} K_{D_\infty }(v)\le (1+\epsilon )K_{D_n}(v). \end{aligned}$$

### Proof

Fix an Hermitian metric on $TD_\infty$. The result immediately follows if we prove (2) for all $v\in \pi ^{-1}(H)$ such that $\Vert v\Vert =1$. Assume by contradiction that there exist $H\subset D_\infty$ compact, $\epsilon >0$, and $n_k\rightarrow +\infty$, $v_k\in \pi ^{-1}(H)$ such that $\Vert v_k\Vert =1$ and

$$\begin{aligned} K_{D_\infty }(v_k)> (1+\epsilon )K_{D_{n_k}}(v_k). \end{aligned}$$

We can assume that $v_k\rightarrow v_\infty \in \pi ^{-1}(H)$. Let $\phi _k:\mathbb {D}\rightarrow D_{n_k}$ be a holomorphic map such that $\phi _k(0)=\pi (v_k)$ and $\alpha _k\phi _k'(0)=v_k$, where $\alpha _k\le (1+\epsilon )^{1/2}K_{D_{n_k}}(v_k)$. In particular, $\alpha _k\le (1+\epsilon )^{-1/2}K_{D_{\infty }}(v_k)$ and hence, $\alpha _k$ is uniformly bounded in *k*. We may, therefore, assume that $\alpha _k$ converges to a limit $\alpha$ as $k\rightarrow +\infty$.

Since *M* is taut and $\phi _k(0)\in H$, we can assume that the sequence $\{\phi _k\}_k$ converges uniformly on compact sets to a holomorphic map $\phi :\mathbb {D}\rightarrow M$, which satisfies the identity $\alpha \phi '(0)=v_\infty$. Using property (i), we have $\phi (\mathbb {D})\subset \overline{D}_\infty$. Since $D_\infty$ has simple boundary in *M* it follows from $\phi (0)=\pi (v_\infty )\in D_\infty$ that $\phi (\mathbb {D})\subset D_\infty$. Finally using the definition of the Kobayashi–Royden metric, we have

$$\begin{aligned} K_{D_\infty }(v_\infty )\le \alpha \le \lim _k(1+\epsilon )^{-1/2}K_{D_\infty }(v_k)=(1+\epsilon )^{-1/2}K_{D_\infty }(v_\infty ), \end{aligned}$$

which is a contradiction. $\square$

### Proof of Proposition 26

The uniform convergence on compact subsets of the Kobayashi–Royden metric follows from Lemmas 27 and 28. We now prove the local uniform convergence of the Kobayashi distance. In what follows, we denote by $\ell _M(\gamma )$ the Kobayashi–Royden length of a curve $\gamma$ on the manifold *M*.

Let $H\subset D_\infty$ be a compact set, and set $R:=\textrm{diam}(H)$. Given $p,q\in H$ and $\epsilon \in (0,1)$, let $\gamma :[0,1]\rightarrow D_\infty$ be a piecewise $C^1$ curve joining *p* with *q* and satisfying $\ell _{D_\infty }(\gamma )\le k_{D_\infty }(p,q)+\epsilon$. Fix $o\in H$. Then, for all $t\in [0,1]$,

$$\begin{aligned} k_{D_\infty }(o,\gamma (t))\le & {} k_{D_\infty }(o,p)+k_{D_\infty }(p,\gamma (t))\\ {}\le & {} R+\ell _{D_\infty }(\gamma )\le R+k_{D_\infty }(p,q)+\epsilon \le 2R+1, \end{aligned}$$

i.e., the support of $\gamma$ is contained in $\overline{B_{D_\infty }(o,2R+1)}$ which is a compact subset of $D_\infty$ by the completeness of $D_\infty$. By Lemma 27, there exists *N* such that for all $n\ge N$ and for all $v\in \pi ^{-1}(\overline{B_{D_\infty }(o,2R+1)})$ we have $K_{D_n}(v)\le (1+\epsilon )K_{D_\infty }(v),$ which implies $\ell _{D_n}(\gamma )\le (1+\epsilon )\ell _{D_\infty }(\gamma )$. Hence,

$$\begin{aligned} k_{D_n}(p,q)\le & {} \ell _{D_n}(\gamma )\le (1+\epsilon )\ell _{D_\infty }(\gamma )\le (1+\epsilon )(k_{D_\infty }(p,q)+\epsilon )\\ {}\le & {} k_{D_\infty }(p,q)+O((1+R)\epsilon ). \end{aligned}$$

In particular,

3 $$\begin{aligned} k_{D_n}(p,q)=O(1+R) \end{aligned}$$

for $$n\ge N$$.

For the converse, notice that by (ii) *H* is eventually contained in the domains $D_n$. Given $p,q\in H$ and $\epsilon \in (0,1)$, let $\gamma _n:[0,1]\rightarrow D_n$ be a piecewise $C^1$ curve joining *p* with *q* and satisfying $\ell _{D_n}(\gamma _n)\le k_{D_n}(p,q)+\epsilon$. Fix $o\in H$ and define

$$\begin{aligned} t_n:=\sup \{t\in [0,1]:\gamma _n([0,t])\subset B_{D_\infty }(o,2R)\}. \end{aligned}$$

We have that $k_{D_\infty }(p,\gamma _n(t_n))\ge k_{D_\infty }(p,q)$. Indeed, this clearly holds if $t_n=1$. If $t_n<1$, then $k_{D_\infty }(o,\gamma _n(t_n))=2R$ and thus

$$\begin{aligned} k_{D_\infty }(p,\gamma _n(t_n))\ge k_{D_\infty }(o,\gamma _n(t_n))-k_{D_\infty }(p,o)\ge 2R-R=R\ge k_{D_\infty }(p,q). \end{aligned}$$

Since $\overline{B_{D_\infty }(o,2R)}$ is compact, by Lemma 28 there exists *N* such that for all $n\ge N$ and for all $v\in \pi ^{-1}(\overline{B_{D_\infty }(o,2R)})$, we have that $K_{D_n}(v)\ge (1+\epsilon )^{-1}K_{D_\infty }(v).$ Hence,

$$\begin{aligned} k_{D_n}(p,q)+\epsilon&\ge \ell _{D_n}(\gamma _n)\ge \ell _{D_n}(\gamma _n|_{[0,t_n]})\ge (1+\epsilon )^{-1}\ell _{D_\infty }(\gamma _n|_{[0,t_n]})\\&\ge (1+\epsilon )^{-1}k_{D_\infty }(p,\gamma _n(t_n))\ge (1+\epsilon )^{-1}k_{D_\infty }(p,q), \end{aligned}$$

that is

$$\begin{aligned} k_{D_\infty }(p,q)\le (1+\epsilon )(k_{D_n}(p,q)+\epsilon )\le k_{D_n}(p,q)+O((1+R)\epsilon ), \end{aligned}$$

where we used (3). $\square$

Let *W* be a Worm. We call *Barrett’s scaling* the one-parameter group of automorphisms of $Y\times \mathbb {C}$ given by

$$\begin{aligned} \textrm{B}_\lambda :(z,w)\mapsto (z, \lambda w) \qquad (\lambda >0),\end{aligned}$$

which played a key role in [4, Sect. 4].

For all $n\ge 1$ we set $D_n:= \textrm{B}_n(W)$, $D_\infty := W_{\textrm{in}},$ and $M:= W_{\textrm{out}}.$

### Remark 29

Properties (i) and (ii) of Proposition 26 are satisfied in this case.

### Lemma 30

The domain $W_{\textrm{in}}$ has simple boundary in $W_{\textrm{out}}$.

### Proof

Let $\varphi :\mathbb {D}\rightarrow W_{\textrm{out}}$ be a holomorphic map such that $\varphi (\mathbb {D})\subset \overline{W}_{\textrm{in}}$. Assume that there exists $\zeta _0\in \mathbb {D}$ such that

$$\begin{aligned} (z_0,w_0):=\phi (\zeta _0)\in \partial W_{\textrm{in}}. \end{aligned}$$

Clearly $z_0\in \partial X_{\textrm{in}}$. If $\pi _1:X_{\textrm{out}}\times \mathbb {C}\rightarrow X_{\textrm{out}}$ denotes the projection to the first variable, then $\pi _1\circ \phi :\mathbb {D}\rightarrow X_{\textrm{out}}$ is a holomorphic function with image contained in $\overline{X}_{\textrm{in}}$ and such that $(\pi _1\circ \phi )(\zeta _0)\in \partial X_{\textrm{in}}$, hence by the open-mapping theorem $\pi _1\circ \phi$ is constant. Thus, $\phi (\mathbb {D})\subset \partial W_{\textrm{in}}$.$\square$

We are finally able to prove our main theorem.

### Proof of Theorem 1

By contradiction, assume that there exists $\delta \ge 0$ such that for each $o,x,y,z\in W$ we have

$$\begin{aligned} \min \{(x|y)^{k_W}_o,(y|z)^{k_W}_o\}-(x|z)^{k_W}_o\le \delta . \end{aligned}$$

Now since for all $n\ge 1$, the Barrett’s scaling $B_n$ is an isometry between *W* and $B_n(W)$ we have, for each $o,x,y,z\in B_n(W)$,

$$\begin{aligned} \min \{(x|y)^{k_{B_n(W)}}_o,(y|z)^{k_{B_n(W)}}_o\}-(x|z)^{k_{B_n(W)}}_o\le \delta . \end{aligned}$$

By Proposition 26, we have, for all $o,x,y,z\in W_{\textrm{in}}$,

$$\begin{aligned}{} & {} \min \Big \{(x|y)^{k_{W_{\textrm{in}}}}_o,(y|z)^{k_{W_{\textrm{in}}}}_o\Big \}-(x|z)^{k_{W_{\textrm{in}}}}_o\\ {}= & {} \lim _{n\rightarrow +\infty }\min \Big \{(x|y)^{k_{B_n(W)}}_o,(y|z)^{k_{B_n(W)}}_o\Big \}\\{} & {} \quad -(x|z)^{k_{B_n(W)}}_o\le \delta . \end{aligned}$$

Thus, $W_{\textrm{in}}$ is Gromov hyperbolic, which contradicts Corollary 24. $\square$

## References

[1] Adachi M, Yum J (2021). Diederich–Fornæss and Steinness indices for abstract CR manifolds. J. Geom. Anal..

[2] Balogh Z, Bonk M (2000). Gromov hyperbolicity and the Kobayashi metric on strictly pseudoconvex domains. Comment. Math. Helv..

[3] Baouendi MS, Ebenfelt P, Rothschild LP (1999). Real Submanifolds in Complex Space and Their Mappings.

[4] Barrett D (1992). Behavior of the Bergman projection on the Diederich–Fornæss worm. Acta Math..

[5] Barrett D (1998). The Bergman projection on sectorial domains. Contemp. Math..

[6] Bedford E, Fornæss JE (1978). A construction of peak functions on weakly pseudoconvex domains. Ann. Math..

[7] Bracci, F., Gaussier, H., Zimmer, A.: The geometry of domains with negatively pinched Kaehler metrics. J. Differ. Geom. (2018). arXiv:1810.11389

[8] Bridson, M., Haefliger, A.: Metric Spaces of Nonpositive Curvature, Grundlehren der Mathematischen Wissenschaften [Fundamental Principles of Mathematical Sciences], vol. 319. Springer, Berlin (1999)

[9] Dall’Ara, G., Mongodi, S.: The core of the Levi distribution. arXiv:2109.04763

[10] Dall’Ara, G., Mongodi, S.: Remarks on the Levi core. arXiv:2305.17439

[11] Diederich K, Fornæss JE (1977). Pseudoconvex domains: an example with nontrivial Nebenhülle. Math. Ann..

[12] Docquier F, Grauert H (1960). Levisches Problem und Rungescher Satz für Teilgebiete Steinscher Mannigfaltigkeiten. Math. Ann..

[13] Fiacchi M (2022). Gromov hyperbolicity of pseudoconvex finite type domains in $$\mathbb{C} ^{2}$$. Math. Ann..

[14] Gaussier H (1999). Tautness and complete hyperbolicity of domains in $$\mathbb{C} ^{n}$$. Proc. Am. Math. Soc..

[15] Gaussier H, Seshadri H (2018). On the Gromov hyperbolicity of convex domains in $$\mathbb{C} ^{n}$$. Comput. Methods Funct. Theory.

[16] Kobayashi, S.: Hyperbolic Complex Spaces. Grundlehren der Mathematischen Wissenschaften [Fundamental Principles of Mathematical Sciences], vol. 318. Springer, Berlin (1998)

[17] Krantz S, Peloso M (2008). Analysis and geometry on worm domains. J. Geom. Anal..

[18] Noell A (2008). Peak points for pseudoconvex domains: a survey. J. Geom. Anal..

[19] Zimmer A (2016). Gromov hyperbolicity and the Kobayashi metric on convex domains of finite type. Math. Ann..

